# Neuritis of the Peripheral Nerves

**Published:** 1891-05-16

**Authors:** J. Michell Clarke

**Affiliations:** Assistant Physician Bristol General Hospital


### Neuritis of the Peripheral Nerves.

By J. Michell Clarke, M.A., M.B., M.R.C.P., Assistant Physician Bristol General Hospital.

I.

Under this head I propose to consider some cases in which one or more of the nerves of one limb were affected with inflammation; cases which, from the limitation of the mischief, do not come under the head of multiple neuritis. The causes of neuritis are very various, and the disorder generally occurs in persons of adult age, and runs a shorter or longer course according to its severity. In neuritis from poisons circulating in the blood, such as lead, alcohol, and the diphtheritic poison, the distribution is more general, and cases of these affections will, therefore, not be included.

Taking first the upper extremity, the following is an instance of neuritis of some of the roots of the brachial plexus. The patient, a thin, anæmic man, æt. 50, a warehouseman, complained of severe pains in the right arm and shoulder, with wasting and paralysis of the limb. He had always been healthy until five years previously, when he had a severe attack of bronchitis, since then he had been liable to milder attacks of bronchitis with each recurring winter. The illness began six months previous to admission with aching pains in the right side of the neck, often agonising in their intensity; they gradually extended to the shoulder, and movement of the limb caused much pain. Four months after the onset the pain had reached to the elbow, and there was by this time marked wasting of the shoulder muscles. During the whole six months he was incapacitated from work. When he first came under observation the pains in the neck had ceased, but still continued in the shoulder and arm, and were especially felt behind the inner condyle of the humerus as if his "funny bone" had been struck. The shoulder and elbow joints were very tender to the touch, and there was hyperæsthesia of the skin over the shoulder and deep-seated tenderness in the muscles of the shoulder and arm. Pressure over the brachial plexus, over the ulnar and median nerves, and at Erb's point, *i.e.*, a point lying in a line drawn from the seventh cervical spine to the sterno-clavicular articulation, at a distance of 1·5 centimetres from the edge of the trapezius. There was no redness or swelling of the joints, but movements at the shoulder and elbow were weak and limited in range, and caused an aggravation of the pain. The dynamometer gave 35 kilogrammes for the left, and six for the right hand. The deltoid, supra- and infra-spinatus, were decidedly wasted; the triceps, biceps, and supinator longus showed less marked wasting; the muscles of the forearm and hand were not wasted, but were weak and flabby. The muscles generally of the limb contracted too readily on direct percussion over them. Electrical examination gave reaction of degeneration in supra-spinatus and infra-spinatus; increase to constant and slight diminution to faradic current in deltoid; increased reaction to both forms of current for biceps, triceps, supinator longus, pectoralis major, and latissimus dorsi. There was some loss of sensation over the shoulder joint, and over the posterior surface of the arm and forearm to within two inches of the wrist joint; this loss was made out for all forms of sensation. The two points of the æsthesiometer were recognised only at a distance of 5 cc from each other over the shoulder, 3½ cc back of arm, and 3 cc back of forearm. There was a patch of hyperæsthesia about the size of a florin over the base of the acromion process of the scapula and another of the same size over the inner condyle of the humerus.

The patient first came under treatment on July 22nd; he was given cod-liver oil, iron, and arsenic internally, and applications of the constant current were made for 10-15 minutes every day; the positive pole placed at Erb's point, and the negative gently stroked over the affected muscles. Under this treatment pain was relieved, the muscles gained strength and volume, and the dynamometer registered 26 kilos for right hand on September 22nd. The cause of the neuritis in the absence of any predisposing circumstance in the family and personal history was doubtful till October 1st, when the patient had an attack of acute rheumatism in the left wrist joint, which made it probable that we had to deal with a rheumatic affection of the nerve sheaths. He now took Salol gr. xx every four hours, and subsequently iodide of potash in the ordinary rheumatic mixture. He made rapid progress; on November 4th the dynamometer registered 34 kilos, the muscles had filled up, and appeared of good strength, and he was dismissed cured on November 19th; the duration of the illness being thus 11 months; probably he would have recovered more quickly had he come under treatment earlier.

The next case was a milder one of the same kind. The patient was a washerwoman, æt. 65, who attended on February 24th, 1890, for pains in right arm and shoulder of six months' duration. The pains were worse at night, and were "gnawing" in character. She was losing strength in the arm, and had noticed some wasting of the shoulder. The chief movements interfered with were abduction of the arm, and the action of putting it behind her back. She had always been temperate, and with the exception of an attack of nephritis twenty years previously had suffered from no illness. She was anæmic; the apex-beat of the heart was strong, a little outside the nipple line in the fifth space, and the second sound in the aortic area was accentuated; the urine was, however, normal. There was no evidence of rheumatism in any joint, nor any loss of sensation over the limb. Tender spots existed at Erb's point, over the external and internal lateral ligaments of the elbow, and at the insertion of the deltoid. The deltoid and spinati muscles were paralysed and somewhat wasted; the biceps was affected in a less degree, and the supinator longus was still less involved; the other muscles were normal, except that the pectorales on both sides appeared weak and flabby, which was put down to her feeble state of health. The above muscles reacted too readily to direct percussion. Electrical examination gave for nerve at Erb's point and for deltoid muscle, increased reaction to faradism. To galvanism, estimated in Leclanché cells:—

| | | | | |
|---|---|---|---|---|
| Nerve, R. side............ | KCC = ACC 10 | AOC 12 | | |
| ,, L. side............ | KCC 10 | ACC 14 | | |
| Deltoid muscle, right... | KCC = ACC 8 | AOC 10 | KOC 16 | |
| ,, left ... | KCC 12 | ACC = AOC 18 | | |

There was thus increased reaction to galvanism with some qualitative change. Under treatment with applications of galvanism in the manner above indicated, and with arsenic, iron, and cod-liver oil internally, she made a good recovery, and was quite well on April 18th.

Both these cases in the main showed the distribution of a paralysis of the upper extremity first described by Erb, in which the muscles affected are the deltoid, the spinati, biceps, brachialis anticus, and supinator longus. This group of muscles can be put into action by electrical stimulation at the point above-mentioned between the scaleni, and their paralysis seems to be brought about by a neuritis at this point affecting the fifth and sixth cervical nerves. In the first case the triceps was affected as well, and the neuritis was more intense than in the second. The causes of this paralysis are injury, neuritis, pressure from a tumour, and occasionally injury during birth. Where the arm has been kept completely at rest for some time on account of pain, the other muscles of the limb become weak and flabby from disuse, and the paralysis at first sight seems more extensive than is really the case; but the wasting and the change in the electrical reactions of the muscles chiefly affected make the diagnosis clear. The pain is generally attributed by the patient to rheumatism in the shoulder joint, and the loss of power put down to the pain caused by movement, but the absence of the ordinary signs of joint affection and the predominant muscular wasting will prevent any doubt on this score. Tender spots are generally to be found at Erb's point, at the insertion of the deltoid, over the coracoid process, and over the external and internal lateral ligaments of the elbow. As in cases of any severity the duration of the illness extends over some months, it is well to begin gentle movement of the shoulder joint as early as the pain permits, otherwise adhesion of the joint surfaces takes place, and gives rise to further pain and trouble in the later stages.

